# Should Gene Editing Be Used to Develop Crops for Continuous-Living-Cover Agriculture? A Multi-Sector Stakeholder Assessment Using a Cooperative Governance Approach

**DOI:** 10.3389/fbioe.2022.843093

**Published:** 2022-02-25

**Authors:** Nicholas R. Jordan, Jennifer Kuzma, Deepak K. Ray, Kirsten Foot, Madison Snider, Keith Miller, Ethan Wilensky-Lanford, Gifty Amarteifio

**Affiliations:** ^1^ Agronomy and Plant Genetics, University of Minnesota, Saint Paul, MN, United States; ^2^ School of Public and International Affairs, Genetic Engineering and Society Center, NC State University, Raleigh, NC, United States; ^3^ Institute on the Environment, University of Minnesota, Saint Paul, MN, United States; ^4^ Department of Communication, University of Washington, Seattle, WA, United States; ^5^ Terraluna Collaborative, Minneapolis, MN, United States

**Keywords:** gene editing, agricultural diversification, multi-stakeholder, governance, cover crops

## Abstract

Continuous-living-cover (CLC) agriculture integrates multiple crops to create diversified agroecosystems in which soils are covered by living plants across time and space continuously. CLC agriculture can greatly improve production of many different ecosystem services from agroecosystems, including climate adaptation and mitigation. To go to scale, CLC agriculture requires crops that not only provide continuous living cover but are viable in economic and social terms. At present, lack of such viable crops is strongly limiting the scaling of CLC agriculture. Gene editing (GE) might provide a powerful tool for developing the crops needed to expand CLC agriculture to scale. To assess this possibility, a broad multi-sector deliberative group considered the merits of GE—relative to alternative plant-breeding methods—as means for improving crops for CLC agriculture. The group included many of the sectors whose support is necessary to scaling agricultural innovations, including actors involved in markets, finance, policy, and R&D. In this article, we report findings from interviews and deliberative workshops. Many in the group were enthusiastic about prospects for applications of GE to develop crops for CLC agriculture, relative to alternative plant-breeding options. However, the group noted many issues, risks, and contingencies, all of which are likely to require responsive and adaptive management. Conversely, if these issues, risks, and contingencies cannot be managed, it appears unlikely that a strong multi-sector base of support can be sustained for such applications, limiting their scaling. Emerging methods for responsible innovation and scaling have potential to manage these issues, risks, and contingencies; we propose that outcomes from GE crops for CLC agriculture are likely to be much improved if these emerging methods are used to govern such projects. However, both GE of CLC crops and responsible innovation and scaling are unrefined innovations. Therefore, we suggest that the best pathway for exploring GE of CLC crops is to intentionally couple implementation and refinement of both kinds of innovations. More broadly, we argue that such pilot projects are urgently needed to navigate intensifying grand challenges around food and agriculture, which are likely to create intense pressures to develop genetically-engineered agricultural products and equally intense social conflict.

## Introduction

Emerging biotechnologies such as gene editing may greatly advance critical frontiers in agricultural development, such as climate resilience or the welfare of resource-poor farmers and increase global food security (Karavolias Nicholas et al., 2021). However, society must also be protected from potential harmful effects—direct or indirect—of these biotechnologies on the environment, human health, or social welfare.

A pilot test of cooperative governance of gene editing (Jordan et al., 2017), applied to crops for continuous-living-cover agriculture, with a particular focus on cover crops, was conducted and is reported on here. Continuous-living-cover (CLC) agriculture integrates multiple crops to create diversified agroecosystems in which soils are continuously covered by living plant cover across time and space. Cover crops are an important element of CLC agriculture. By definition, cover crops are grown on farmland that would otherwise be fallow; these crops can enhance soil, water, and biodiversity in agricultural ecosystems by a wide range of mechanisms (Basche and DeLonge, 2017). So-called “cash cover crops” are a subset of cover crops which produce marketable agricultural commodities. In exploring the prospect of such applications of gene editing, this pilot project addressed matters of broad and global interest, as crops for CLC agriculture are widely seen as fundamental to progress on regeneration of degraded soils, which in turn is critical to sustaining agriculture productivity, water, biodiversity, and to climate adaptation and mitigation.

The initial stage of the cooperative governance pilot was a multi-sector, multi-stage deliberative process (Jordan et al., 2017). These deliberations included assessment of rewards and risks from potential applications of gene editing to cover crops, cash-cover crops, and other crops of particular value for CLC agriculture in temperate zones—such as the US Midwest region—where annual row crops now predominate. Deliberations also addressed governance, i.e., how such applications might be governed to manage inherent rewards and risks.

A key premise of the deliberative process is that any effort to use gene editing as a means for developing crops for CLC agriculture would succeed at scale only with multiple pillars of support, including development of markets for such crops, provision of finance, supportive policy, and social cohesion and collective action (per Herrero et al., 2020). Therefore, we recruited actors and stakeholders relevant to such sectors (e.g., markets, finance, policy, NGOs, think-tanks, farmers, trade organizations, industry, government, and academe) into the pilot project. We therefore consider our assessment of these applications of gene editing to be pragmatic, in the sense of being informed by the views and perceptions of actors and stakeholders that would be central to any effort to develop such crops *via* gene editing. Our project appears to be a relatively unique effort to convene and support a multi-stakeholder deliberative process around applications of GE to sustainable development of agriculture (see also Lotz et al., 2020). Implementation of such processes has been very limited, despite many calls for their use in governance of emerging biotechnologies (Kuzma, 2016; NAS, 2016; Jordan et al., 2017; Jasanoff and Hurlburt, 2018; Kofler et al., 2018; Montoliu et al., 2018; Resnik, 2018).

We note that at present, development and scaling of gene-edited crops of any sort is in early days. Certainly, assessment of the merits of gene editing should address presently evident risks and opportunities. However, any assessment of gene editing as a means of developing cover crops and cash cover crops must also be prospective and anticipatory, given the lack of actual experience. In particular, we suggest that it is necessary to enlarge the scope of assessment to encompass ***feasible methods for identification, assessment, and management of emerging rewards, risks***, ***and societal impacts of gene editing applied to the crops of interest, as such applications go forward.*** We can anticipate, based on the history of scaling of innovations (Herrero et al., 2020; Wigboldus et al., 2020) that additional rewards, risks, and impacts will indeed emerge as the result of technological development, crop applications, scaling of resultant crops, and growing understanding of biophysical and social effects of these crops. Moreover, it is clear that broad stakeholder support for such applications is contingent on how emerging effects of applications are identified, assessed and managed (e.g., Gordon et al., 2021). Therefore, methods and capacities for managing the inherent dynamics and complexities of rewards, risks, and societal impacts are an important aspect of the use of gene editing to develop cover crops and cash cover crops.

Below, we outline key motivations for our pilot cooperative governance project, describe its initial deliberative phases, and report findings from interviews and workshops with participants and other actors, in the context of recent developments in governance of gene editing.

### Motivations for Cooperative Governance of Gene Editing Applied to Crops for CLC Agriculture

#### Global Need for Diversified, Broadly-Regenerative Agriculture

Major transitions are needed in agriculture to create a broadly-regenerative agriculture, i.e., an agriculture that can remedy pervasive degradation of soil, water, and biodiversity, provide climate-change adaptation and mitigation benefits, reduce diet-related health problems, and address inequity and injustice in agriculture and food systems (HLPE, 2019; Willett et al., 2019; Klerkx and Begemann, 2020; Rockström et al., 2020; Steiner et al., 2020). Diversification of current farm production systems appears fundamental to meeting these goals. Through a wide range of mechanisms, diversification can improve the condition of soil, water, and biodiversity resources (Lin, 2011; Kremen and Miles, 2012; Bowles et al., 2020; Tamburini et al., 2020), enable climate-change adaptation and mitigation, and support dietary shifts to lower the carbon intensity of human diets. Diversification also creates opportunities to enhance equity and other aspects of social sustainability, if socio-economic interventions that address these aspects are encompassed in diversification initiatives.

### Diversification *via* Continuous Living Cover Agriculture

Several major diversification projects in agriculture rest on a concept of continuous living cover of farmland (Basche and DeLonge, 2017). These projects and initiatives are being implemented globally under a variety of banners, including “conservation agriculture,” “soil health,” and “regenerative agriculture,” as the latter is most commonly framed (Lal, 2020), and have been strongly supported and advocated by public, private, and advocacy sectors. The common theme is regeneration of degraded soils as a means of enhancing agriculture productivity, water resources, biodiversity, and climate adaptation and mitigation. The essence of these projects is the design and scaling of agroecosystems that minimize soil disturbance and maximize the coverage of farmland with living crop-plant cover across the annual cycle (Jayaraman et al., 2021). In this concept of agroecosystem design and management, diversification is inherent because a range of crop and crop types (e.g., both annual, perennial) is necessary to achieve CLC across farmland and across the annual cycle. There is considerable evidence that agroecosystems based on CLC can support regeneration and provide climate adaptation and mitigation (Asbjornsen et al., 2008; Asbjornsen et al., 2014; Landis, 2017; Schulte et al., 2017; Brandes et al., 2018; King and Blesh, 2018; Burchfield et al., 2019).

### CLC Agriculture Depends on Development of New Crops

Unfortunately, CLC agroecosystems often do not offer attractive short-term returns on investments (i.e., favorable cost/benefit ratios) (Plastina et al., 2020), or otherwise are economically feasible for only a subset of farmers (Giller et al., 2009). The unfavorable economics of CLC agroecosystems largely result from functional limitations of CLC crops—i.e., crops that can be used to increase continuous living cover in these agroecosystems. For example, in temperate-zone agriculture, fallow-season cover crops have received much attention in recent years. Such crops are planted in a fallow season after harvest of predominant crops (often summer annual crops such as maize and soybean) to conserve soil, water, and biodiversity, or to regenerate these elements of agroecosystems when their condition is degraded. By definition, these cover crops are not harvested for any marketable agricultural commodity. At the present time, use of such cover crops remains very limited in some major agricultural regions, such as the Midwest of North America (Rundquist and Cox, 2021), except when high levels of subsidies are provided (Rundquist and Cox, 2021).

Adoption appears to be reduced by functional limitations of these crops, which include limited germination, establishment, and early growth, nitrogen fixation, winter hardiness, slow biomass production and maturity, weed suppression, challenges in transition from cover crop to a subsequent crop, and limited seed production. Historically, cover crop breeding efforts have been very modest compared to dominant crops (Wayman et al., 2017); more comprehensive breeding programs are critically needed to reduce these functional limitations (Runck et al., 2020). In practice, these limitations are manifested as economic costs to farming operations that use cover crops.

One fundamental strategy for improving these economics is the development of “cash cover crops,” as mentioned above. By definition, such crops provide both the agroecological benefits of cover crops, and yield valuable products for which scalable markets exist. A prime example of such a crop is camelina (*Camelina* s*ativa*), which can serve as a cover crop while also showing high potential for many market opportunities (Zanetti et al., 2021). Most crops with high potential for such a dual-function role are novel or previously minor crops, and like camelina, many aspects of these crops need development to realize their potential. Specifically, development of these crops requires improved understanding of their genetics, genomics, and breeding; agronomic methods; agroecological interactions and effects; supply-chain infrastructure; and processing and product manufacturing. Therefore, a broad and robust program of crop breeding and development is needed to realize the potential of CLC agriculture. One such a program is the Forever Green Partnership, a broadly-based multi-sector/cross-scale collaboration that is working to develop CLC agriculture (Forever Green Partnership, 2021). Members of the Forever Green Partnership were the initial organizers of the pilot cooperative governance project, motivated by the project’s interest in possible applications of gene editing in its development of CLC crops.

### Crop-Breeding Strategies for Rapid Development of Crops for CLC Agriculture and Other Forms of Diversification

Integrative crop-breeding strategies are emerging for rapid genetic advancement of novel, previously minor, and “orphan” crops that can enable CLC agriculture and other forms of diversification. These strategies (e.g., Guilenque et al., 2020) integrate conventional breeding methods with genomic approaches that are based on DNA-sequence data obtained by the advent of rapid, inexpensive sequencing of whole genomes. Strategies can also include participatory breeding methods, in which farmers join as integral members of the breeding program (Runck et al., 2014). For example, integrative breeding programs are being applied to a range of novel legume crops (Jiang, 2021).

The emerging technologies of gene editing may substantially accelerate development of new crops through integrative crop-breeding strategies. In particular, recent applications of gene editing to orphan crops in the genus *Physalis* suggest potential for rapid improvement in functional traits key to widespread commercialization. Crop breeders (Lemmon et al., 2018) envision that a range of orphan crops, currently important to smallholder agriculture in various regions globally (e.g., teff, grain amaranth, and cowpea) might be “catapulted into mainstream agriculture” by gene editing guided by genomic information from distantly related model crops. Advancing technical and methodological prospects for rapid genetic development of novel crops may provide a means for rapid development of crops for CLC agriculture.

However, public backlash towards 1st generation GM crops, generally used in predominant commodity crops such as maize, necessitates a careful examination of the societal concerns alongside the exploring of benefits of CLC and gene editing (Jordan et al., 2017; Kuzma, 2018). Therefore, our pilot cooperative governance project brought together a multi-sector group to explore the challenges and opportunities associated with CLC agriculture using gene editing.

## Methods

### Cooperative Governance: Initial Deliberative Processes

As noted, we expect that GE applications to develop CLC crops will succeed at scale only with active support from a wide range of societal sectors. Therefore, we recruited actors and stakeholders relevant to such sectors (including markets, finance, policy, NGOs, think-tanks, farmers, trade organizations, industry, government academe) into the pilot project and solicited input from additional subject-matter experts and stakeholders (SMESs) who were not formal participants in the pilot project. Recruitment was done by the lead organizer of the pilot project (Jordan), leveraging his professional networks, seeking participants for these sectors that were interested in joining the pilot project.

**FIGURE 1:**
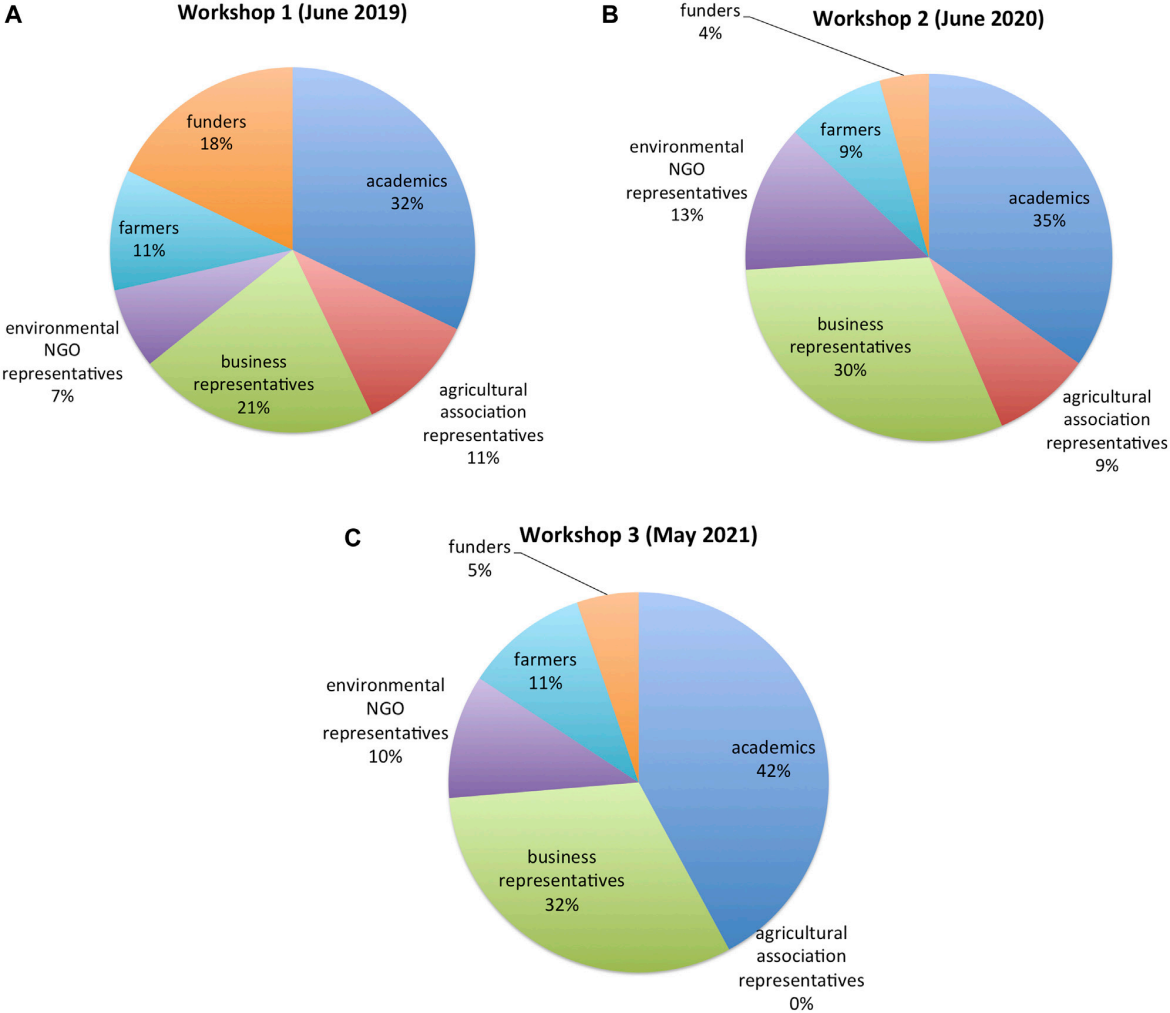
Stakeholder participants in workshops in 2019 **(A)**; 2020 **(B)**; and 2021 **(C)**.

We organized two multi-day deliberative workshop gatherings for the multi-sector cooperative governance network, and a briefer capstone gathering at the end of the initial phase of the project which is ongoing. These workshops engaged a broad range of societal sectors (Figure 1), and were integral to the project’s multi-sector cooperative governance approach to applications of GE to cover crops and “cash cover crops.” The first gathering (2019) was in-person; 2020 and 2021 sessions were virtual, using an online meeting platform. The workshops were designed to facilitate deliberative engagement among project participants to assess the impacts (e.g., economic, environmental, and social) of such applications of gene editing. Importantly, the workshops also considered the possibility of taking collective action to address shared interests in the above governance, and particular options for operationalizing and implementing cooperative governance to manage inherent rewards and risks.

The first workshop was held in-person at the University of Minnesota over 3 days in June 2019. The 28 participants in the 2019 workshop included a wide range of sectors (Figure 1A) and the eight members of the pilot project organizing group, comprising six academics and two project evaluators. The second workshop convening was held online over 2 days in June 2020, with 23 participants from multiple sectors, and the same eight members of the project group (Figure 1B).

The initial workshop was designed to provide baseline knowledge about GE and cover crops, “cash cover crops,” and other crops relevant to CLC agriculture, and to allow participants to exchange perspectives on these topics. The second workshop focused on deliberative discussion of scenarios of application and governance of GE. Scenarios were presented and discussed for two such crops: winter camelina and alfalfa. In small groups, participants discussed anticipated benefits and risks of such applications and several different scenarios for governance of these particular applications were discussed. These governance scenarios were developed by an online Delphi process that solicited participants’ views on options for implementation of cooperative governance. These discussions set the stage for deliberations of a range of contrasting governance scenarios, and of prospects for implementation of one of these scenarios by the project group.

A third capstone workshop (May 2021) reviewed project activities, previewed remaining activities for the initial phase, and proposed a follow-up project for continued piloting of cooperative governance. The 2021 workshop was also held online, with 19 participants from multiple sectors, and the same eight persons from the project group (Figure 1C); its duration was 2 hours.

### Semi-Structured Interviews

**FIGURE 2:**
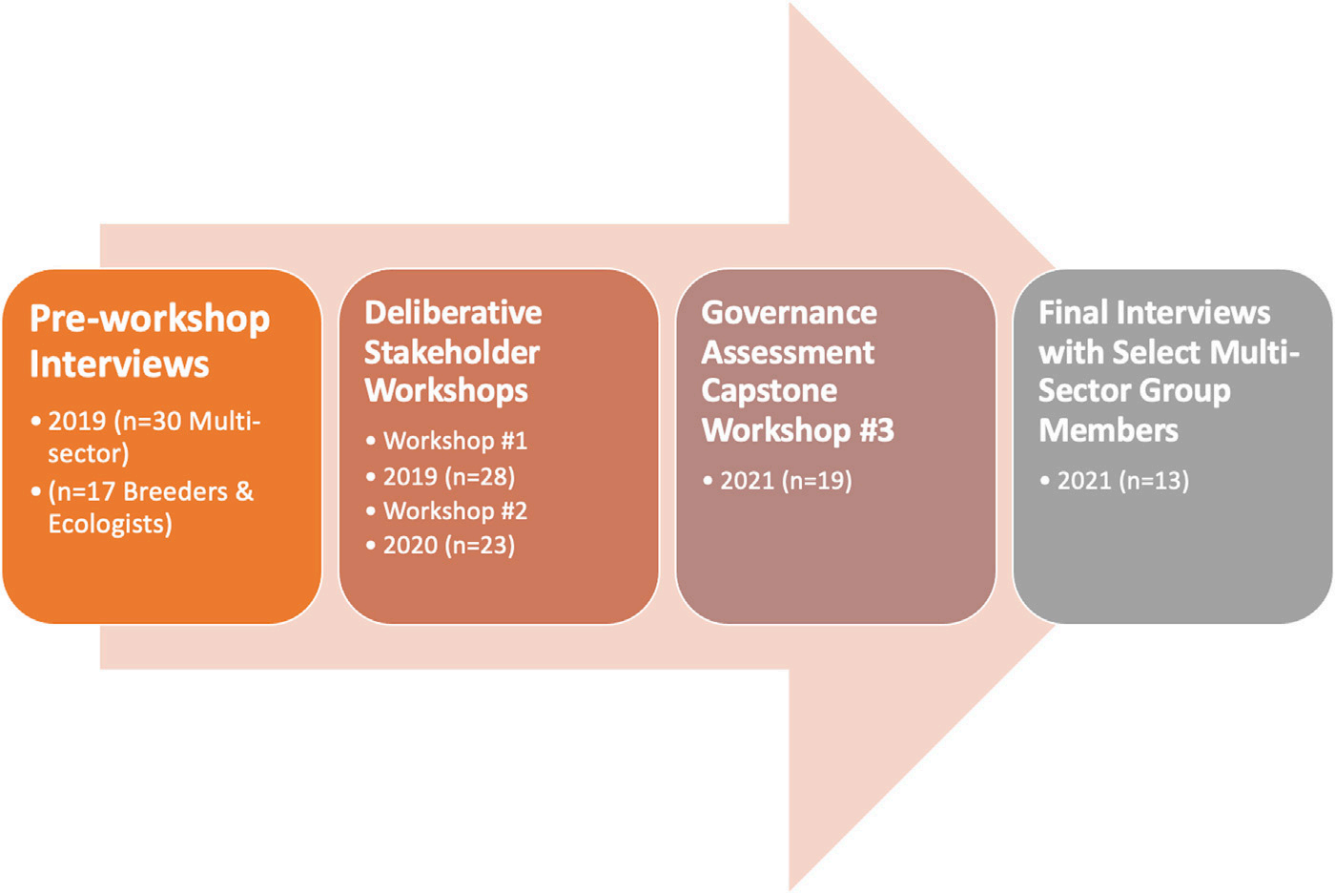
Cooperative Governance Process: Initial Deliberative Stages. Note: for workshop participant affiliations see Figure 1. For interviewee affiliations, see Methods.

We used these interviews to elicit views of pilot-project participants at the project’s inception and after the conclusion of the initial phase of the project. We also interviewed SMES who were not participating in the cooperative governance pilot project. The interviews were conducted prior to and after the three workshops, as shown in Figure 2.

### Initial Interviews at Project Inception

Prior to the first cooperative governance workshop in June 2019, we conducted interviews with 30 participants in the cooperative governance pilot. Four of the initial interview participants were unable to attend the first workshop. The interviews focused on learning how cooperative governance participants viewed the major issues facing agriculture; understood the potential of CLC agriculture; perceived the risks and benefits of genomic editing; and had previously experienced cooperative governance.

The interview participants represented a diverse multi-sector network: 4 came from agricultural associations such as farmer organizations; 12 came from academic institutions engaged in crop, genome, or policy research; 1 came from an environmental NGO; 6 came from business and investment organizations; two were farmers; and 3 came from funding agencies. Two participants had unaffiliated designations.

Interviews were audio-recorded and transcribed for analysis. We used topic areas from the interview protocol instrument to develop a preliminary coding structure in the software MAXQDA, and coded by assigning a specific code to participant responses based on the question and the salient theme of their response. We then reviewed coded segments for each theme to provide comprehensive meaning and determine findings and insights of learning that support the stated purpose of the interview.

### Interviews With Crop Breeders, Crop Geneticists, and Agroecologists

To complement viewpoints elicited in initial interviews with project participants, semi-structured, open-ended interviews were conducted with additional subject-matter experts, i.e., 17 geneticists and crop breeders, whose affiliations spanned academia, research institutes, and private companies; all were involved with development with crops for CLC agriculture. Semi-structured, open-ended interviews were also conducted with six agroecologists, whose affiliations included academia and NGOs. A few of these interviewees were full participants in the pilot project, but most were not. The goal of these interviews was to gain additional understanding of the perspectives of these sectors. For geneticists and breeders, interview questions addressed their views on the merits and drawbacks of GE as a means of developing cover crops, cash cover crops, and other crops for CLC agriculture, relative to alternative means such as “conventional” plant breeding, and the importance and urgency of developing such crops. For agroecologists, questions also queried views on the merits and drawbacks of GE as a means of developing crops for CLC agriculture, relative to alternative means such as “conventional” plant breeding. In addition, we elicited comment on anticipated environmental effects—desirable and undesirable—of widespread adoption of cover crops, cash cover crops, and other crops for CLC agriculture. Finally, we queried views on effects of such adoption of social sustainability. Interviewees were identified through professional networks and on the basis of interest in crops for CLC agriculture. All invitees agreed to an interview. Questions were provided in advance. All interviews were conducted by video or audio call and audio-recorded. Recordings were analyzed to summarize responses to questions, and frequently-expressed themes were identified and compiled.

### Post-Workshop Interviews

These interviews explored participants’ perspectives on GE as applied to crops for CLC agriculture, and how those perspectives have shifted over the past 2 years, during the duration of the pilot project. All 26 individuals that participated in the final cooperative governance workshop in 2021 (Figure 1C) were invited to a post-workshop interview. The 13 final interviews came from five different sectors: one farmer; two from agricultural organizations; three from environmental NGOs; three from academic institutions; and four from agricultural businesses.

These interviews encompassed several topics. First, we invited participants to talk about their perspectives on GE in regenerative agriculture. Second, we invited the participants to evaluate their experience in the pilot test of cooperative governance of GE. We also invited participants to express and discuss interest in participating in several options for a future second phase of the pilot test. Once the interviews were completed, the recorded files were transcribed for analysis as described in the pre-workshop interview section.

## Results and Discussion

### Merits and Demerits of GE for Developing Crops for CLC Agriculture

We summarize (Table 1) themes from discussions of the multi-sector group and interviews subject-matter experts and stakeholders (SMESs) who were not participating in the cooperative governance pilot project. These themes encompassed views, reported below, on technical merits, agroecological effects, and societal impacts associated with CLC crop development by GE as compared to alternatives.

**TABLE 1:** Summary of themes from interviews: opportunities and challenges with gene editing and CLC agriculture.

Category	Opportunities (merits)	Challenges (demerits)
Technical Merits/Demerits	• Greater potential to address grand challenges with agriculture using gene editing for CLC	• Need to integrate gene editing with other breeding approaches
	• Realize environmental benefits from CLC agriculture	• Competition with major commodity crops for funding and usage
	• Increased speed and efficiency of CLC crop improvement with gene editing	• Limited understanding of CLC crop genetics and tissue culture for gene editing to work
		• Lack of funding for CLC crop genetics and gene editing
Agroecological Aspects	• Potential to Improve soil quality, biodiversity, and management of crop pests, among other ecosystem services	• Potential ecosystem risks from CLC crops and gene editing
	• With more rapid development of CLC crops through gene editing, more could be evaluated for ecosystem risks and benefits to select best for environment and diversified farming systems	• Possibility that CLC crops become monocultures if incentives for certain cash cover crops
		• Gene flow from CLC gene edited crops to wild relatives
		• Greater environmental movement of companion herbicides or pesticides used with gene edited cover crops
Societal Impacts	• Improvement of ecosystems and agricultural resilience	• R&D investments can be risky due to uncertain scalability
	• Possibility of developing more inclusive governance models around CLC agriculture and gene editing given early stages of field	• Costs of licensing of technology and regulatory compliance
	• Opportunity to increase public support through public and consumer value of CLC and gene editing	• Fear of over-commodification of CLC crops with gene editing due to investment need
		• Potential for greater inequality among farmers and harm to organic farmers
		• Fear of public opposition to gene editing in CLC agriculture

### Technical Aspects of Plant Breeding and Germplasm Development

In our interviews with geneticists and breeders working on developing crops for regenerative agriculture, they were generally keen to use GE technology, because they saw much at stake. Specifically, our interviewees agreed that rapid development of new CLC crops was highly important, because of the large potential benefits of expanding CLC agriculture. Broadly, our interviewees were concerned about “‘*missing out on environmental benefits*,” such as reducing soil erosion, nutrient pollution of water, and coastal hypoxia. One participant suggested that without GE we would not be able to “*change the trajectory*” of “*grand challenges in agriculture/food/environment*.” The key issue is that these geneticists/breeders consider new CLC crops—or existing crops that have received relatively little breeding effort, such as winter camelina—to need substantial genetic improvement, and therefore interrelated considerations of cost, speed, and efficiency of alternative breeding methods are of paramount importance. One of our interviewees noted that “*we could make a theoretical calculation regarding what is the impact of every year of delay on the problems that cover crops can address*.” Without GE we would just “*bumble along with current methods* [and] *maybe won’t achieve key goals*” and “*slow progress or block a viable path to production of a new crop*” with “*desirable traits*” in agronomic or product quality terms. Another described “*getting quite worried about getting the introgression traits and high yield if we don’t use GE*” in cash cover crops.

In particular, geneticists/breeders voiced the need to combine improvement in both polygenic traits (e.g., yield) and key qualitative traits with simpler genetic control (e.g., functional traits of lipids) that are critical to commercialization of new crops. They expressed strong enthusiasm for combining GE of such qualitative traits with other breeding methodologies for polygenic traits. The key point is that simultaneous improvement was needed in both specific traits key to the commercialization of these crops and in a broader range of traits related to general adaptation of these advancing crops. Therefore, the ability to integrate breeding methods was seen as crucial. In general, the breeders and geneticists described the merits of GE in similar terms to those expressed in current accounts of GE (e.g., Jiang, 2021), such as precision of GE relative to alternative methods such as mutagenesis, and unique attributes of editing. They envisioned that, without GE, multiple decades of crop development would be needed to advance their CLC crops to the point of agronomic and commercial viability, whereas they might be able to advance crops to comparable viability within 5–10 years. Crucially, they were skeptical that crop development efforts could be sustained over multi-decade time frames that would be needed without GE, and therefore see GE as essential to development of a broad range of CLC crops. One expert noted that “*regenerative and cover crops have a lot to gain by using GE*” and “*given covid*, *economy, general turmoil—it will be easy for sustainability efforts in agriculture to fall by the wayside*” if “*we don’t use GE* [thereby] *losing momentum*”. Another noted that new crops face “*competition* [with] *major crops—corn*, *soybean—*[that] *are rapidly advancing in yield and productivity*” and “*we could miss our window*” and new “*crops will not be adopted which will be a shame*.” The delay “*may turn into a barrier for companies not to adopt the crop…*”*.*

However, geneticists/breeders emphasized that both knowledge of the fundamental biology of new crops and technical and methodological development are needed to apply GE to the crops they are working on. Specifically, interviewees pointed out that GE requires understanding of ‘functional genomics’ relevant to traits of interest, noting that GE technology “*works well if you know what changes need to be made*.” Importantly, these breeders/geneticists emphasized that applications of GE to CLC crops requires understanding of genetic control of relevant plant phenotypes such as key product quality traits that are important to commercialization of new crops. Interviewees underscored the need for whole-genome sequencing of a reference genome and functional-biology understanding of GE targets as crucial prerequisites for GE applications to CLC crops. Interviewees also pointed out that technical and methodological development are needed to apply GE to some crops they are working on. For example, tissue-culture techniques needed to be developed for certain crops, and these were described as “*works-in-progress*.” These breeders/geneticists also emphasized that considerable time and expense were required for development of both requisite biological and genomic understanding and technical and methodological development, and that resources for development of CLC crops—regardless of particular methods—are quite limited at present. Therefore, investing these limited resources in developing technical and biological knowledge required for GE was perceived as risky by some.

### Agroecological Effects

Participants in our multi-stage deliberative process, and agroecologists working on or strongly concerned with CLC agriculture and associated crops emphasized that development and broad adoption of CLC crops would provide many environmental benefits. The scope of these encompassed the full range of benefits of CLC crops that has been recognized by farmers and researchers, including improvements to soil fertility and quality, reductions in soil erosion and nutrient losses to surface and groundwater, enhancement of biodiversity in agroecosystems and agricultural landscapes, enhancement of total production and production of high-value commodities (e.g., novel sorts of proteins and lipids), farm profitability, and enhanced management of crop pests. Our interviewees underscored the low rates of adoption of conventional cover crops in the Midwest region and the wide range of agroecological problems associated with this lack of continuous living cover across the region. However, they also cautioned that problematic agroecological effects might result from widespread adoption of CLC crops.

Many of these concerns related to contingent effects that might result from any new-crop introduction into established agroecosystems. These include undesirable impacts on disease and arthropod pest dynamics, as might be affected by the practice of “planting green” (planting a crop into undecomposed cover crop residue), or by the persistent presence of cover crop residues soil processes, and nutrient cycling. There was concern that these effects were more likely if there were incentives for production of extensive monocultural stands of CLC crops.

Moreover, they noted potential tradeoffs associated with “cash cover crops,” related to potential conflicts between commodity production and soil, water, and biodiversity conservation effects of cover cropping. Analogous concerns apply to CLC crops in general. Specifically, impacts of nutrient applications to enhance yield of CLC crops raised concerns of enhancing nutrient losses from agroecosystems. Also cover crops can deplete soil moisture in dry years, affecting subsequent crops. Fallow periods of uncovered soil may occur after harvest of cash cover crops.

Also, several concerns that are more particular to GE crops were noted as well. First, the “escape” from farms-- through wind, insect pollen vectors, and seed contamination—of GE plants, GE genes, and GE genomes was noted as a concern. Relevant impacts of such escape include introduction of genetic material into related wild or feral populations, potentially enhancing invasiveness of these populations, and into unedited or non-GMO crops. One expert noted that, previously, “*organic farmers were affected by pollen and pesticide drif*” from GMO crops and “*organic farmers who could no longer sell their produce as organic due to the cross over and were penalized…for something totally out of their control*”.

Another concern relates to externalities such as off-farm movement of pesticides that might be triggered by adoption and scaling of GE crops. For example, extensive adoption GMO crops resistant to the herbicide dicamba has led to major off-farm impacts (Mortensen and Smith, 2020). In non-agricultural ecosystems, plant communities in these ecosystems have been altered by the herbicide, with significant harmful effects on biodiversity conservation in agricultural regions. Herbicide movement has also affected crops that are not resistant to the herbicide.

Essentially, our interviewees emphasized that any novel CLC crop—whether GE or not—might face significant agroecological barriers to scaling as noted above. There was concern that these effects were more likely if there were incentives for production of extensive monocultural stands, as might result if the market value of a CLC crop could be markedly enhanced by use of GE. Importantly, unanticipated and unintended agroecological “downsides” may manifest during the scaling of any new crop. Only ongoing monitoring can detect and manage such effects. Because of these potential limits on scalability, effective diversification of a regional cropping system is likely to require that a number of CLC crops will need to be introduced and evaluated; only some will be scalable in agroecological terms. Therefore, reducing the time and financial costs of CLC crop development is important, which on its face is an argument for using GE to rapidly advance a broad portfolio of CLC crops. On the other hand, it is important to recognize that any particular novel CLC crop may fail to establish at scale for agroecological reasons. This risk must be clear to all parties that invest in these crops, and particularly for the first CLC crops to which GE may be applied with the intent of commercial release of resultant crops.

### Societal Aspects

Participants in the multi-stage process and our other informants were concerned with societal effects of GE of CLC crops. Generally, they saw potential for societal benefit through enhancement of the environmental performance of agroecosystems, and through enhancements in the range and resilience of agricultural production. However, they also were concerned about both procedural and distributive aspects of justice that might be associated with the development and scaling of gene-edited CLC crops.

Regarding the distribution of costs, benefits, and risks of development of GE CLC crop, many informants pointed to the costs of developing CLC crops with GE, considering licensing fees for the editing technologies, costs of regulatory compliance, and the need for capital investments in R&D that are inherently risky because of the uncertain scalability of new CLC crops. All of these factors were viewed as channeling developing of GE crops to well-capitalized private enterprise. Given this pathway for development of CLC crops by GE, our informants were mindful of potential tradeoffs of public goods for private interests. For example, skepticism was raised about applications of GE to produce conventional cover crops, which by definition do not produce marketable commodities. While these crops produce private benefits for farmers, e.g., *via* increasing soil health and other agronomic benefits, they also arguably produce public goods related to soil, water, and biodiversity resources, and climate resilience. Our informants were doubtful that the economic value created by conventional cover crops would motivate private firms to invest in their development by GE. Commodity-producing CLC crops would therefore be the focus of private-sector GE development, raising concerns about tradeoffs between commodity production and other aspects of these crops, as noted above in the discussion of agroecological effects of CLC crops.

Further, it was noted the development of CLC crops will involve wealth creation—because “*cover crops need a whole supply chain and input industry … that is an economic development opportunity*.” The question is: which scales of farmers and what kinds of farmers will benefit from development of CLC crops by GE? Will CLC crop development “*mainly suppor*t *expansion in (large) systems, versus supporting middle and smaller operations?*” Concerns were also expressed about potential production of “product-linked” traits, such as the glyphosate tolerance of “Roundup-Ready” crops, in which the sales of the herbicide glyphosate are inherently promoted by development of such crops. Again, these were seen as opportunities for unwarranted value-capture by the private sector, with potential tradeoffs with public goods. For example, Roundup-Ready crops appear to have degraded a public good—the susceptibility of weeds to glyphosate. Our informants are concerned that if development of GE CLC crops is undertaken mainly by the private sector, such unintended and undesirable consequences may follow.

Informants also raised questions of procedural justice. For example, one informant (an agroecologist) articulated questions of “*who has power in our food system—and how will technologies change power dynamics and take power away from farmers*.” Another expert commented that “*high-value technology out on a field automatically sets up a situation where there is potential social injustice—from the standpoint of someone owning that technology—(with others) being denied the technology—or unable to afford it—or opposed to technology…(which) creates inequality among farmers*.” Another stated “*generally farmers appear to have little power regarding setting prices or structure of agriculture or ability to make changes. It seems that farmers have less and less power; Supply chains and end-use customers—they seem to have outsize power…historically, there has been little consideration of* “*fairness*” *to farmers*.” One informant voiced this concern about justice and trust of public research institutions: “*if the only way these developments can be implemented is by engagement of private sector, and there is a handoff to private sector* … *then this is very damaging to social contracts, including trust in public institutions and science*.” Moreover, “*we do not have precedent for open-source GMO technologies—something along those lines would alleviate potential injustices related to who does and who doesn’t have access to technologies*”.

Project participants and other informants also were concerned about the risk of losing the opportunity to develop and use GE technology for crop development if broad public opposition is aroused. This perceived risk was associated with several different scenarios of concern. First, some participants anticipated that critics of earlier GM crops were prepared to mount strong public campaigns against GE unless they were persuaded that objectionable aspects of those crops and their development will not be repeated in development of gene-edited crops. Secondly, some participants contended that a clear and compelling value proposition to the general public would be critical to avoiding broad public opposition to gene-edited crops. One participant observed “*If we continue to put out products that only bring value back to the farm…*, *I don’t think it's necessarily going to change the paradigm that’s out there, and I guess, what you’re calling as the fear (.) It is a complete fear of the [GE] technology.*” Third, there was concern that mishaps in initial scaling of gene-edited crop—e.g., damage to organic crops by escape of genes from gene-edited crops—would damage the reputation of gene-edited crops in general. Risks associated with such scenario would affect crop developers, investors, and other parties with financial interests in development of particular gene-edited crops, but also may pose the general societal risk of reduced crop development during a time in which agriculture and food systems may face sharply mounting demands related to grand challenges.

### The Use of GE to Develop Crops for Continuous-Living-Cover Agriculture: Social Sustainability and Risk Management Aspects

Below, we turn to a crucial consideration—governance and risk-management aspects of the use of GE to develop crops for CLC agriculture. Based on literature and our interviews with subject-matter experts and stakeholders (SMESs), we identify and discuss existing societal factors that are likely to pose challenges to the adoption, use, and success of gene-edited crops for CLC agriculture. We underscore that these societal factors are existing conditions and circumstances, constituting the current situation and context within which any near-term applications of GE will proceed. In essence, we highlight key aspects of the social “environment”—economic, cultural, political—that will affect adoption, use, and success of applications of GE to CLC crops. In their totality, we judge that this social environment is fraught with barriers and risks affecting successful outcomes from such applications. Therefore, it appears that barriers and risks must be adroitly and adaptively managed if applications of GE are to be successful in advancing the goals of CLC agriculture. It follows that a prospective assessment of the merits of GE for advancing these crops must consider prospects for managing these aspects.

First, we discuss in more detail some of the challenges associated with governance of CLC agriculture and GE (Table 2). Then, we conclude with a discussion about responsible innovation and scaling—based on governance and risk-management mechanisms that are more publicly robust and collaborative (Jordan et al., 2017; Kuzma, 2019; Kuzma and Grieger, 2020)—and how they are likely to be important to managing these barriers and risks, and thus enhancing prospects for successful outcomes in applications of GE to CLC crops.

**TABLE 2:** Summary of governance challenges associated with gene editing and CLC agriculture.

Category	Challenges	Possible remedies
Regulation	• Inability to trace some gene edited crops in CLC agriculture	• Responsible Innovation paradigm and Cooperative Governance models
	• Lack of harmonization for trade with EU	• Ensure robust regulation that is not too costly to small developers
	• Rejection of gene editing by organic agriculture	
	• Over or under-regulating with relation to cost or public confidence, respectively	
Political Economy	• Limited investment in fallow season cover crops generally	• Combine sustainability benefits with production of valuable agricultural commodities to motivate investment in seed cost for farmers and R&D for seed producers
	• Financial risk with investment in CLC gene editing given uncertain scalability	• Develop scalability models
	• Navigating licensing, patents and ownership	• Assistance for small seed developers to navigate intellectual property
Public Acceptance/Social License	• Fear of public opposition to gene editing in CLC agriculture	• Responsible Innovation paradigm and Cooperative Governance models
	• Lack of acceptance of gene editing community that public should have voice in governance	• Better communication about the benefits of gene editing in CLC agriculture
		• Explore voluntary tracking and labeling schemes to ensure consumer choice

### Factors Posing Challenges to Gene-Edited Crops for CLC Agriculture

#### Regulatory Landscapes

Systems of regulation and risk management relevant to gene-edited crops for CLC agriculture vary widely across nations, and create a wide range of challenges to exploration of these crops. In essence, current systems create barriers for poorly-capitalized developers of such crop, while also appearing to some observers as overly lax, arousing concerns for risk management. Finally, the variability of these systems across nations may enhance perceived risks for developers and investors.

Several first-generation GMO crops were regulated by relatively time-consuming regulatory processes. The regulatory impediment to gene-edited CLC crops is much lower in some global regions, but is much more stringent in others. However, even less-stringent regulatory processes may still pose a barrier to crop development efforts that have limited operating capital. Moreover, some civil-society groups consider that less-stringent processes are insufficient to manage public risks associated with GE crops, and these groups may take increasingly oppositional stances in the near future and some are already doing so on the basis of inadequate regulations under the recent 2020 USDA SECURE rule (see Center for Food Safety et al. v National Family Farm Coalition et al. v Vilsack).

Several GM CLC crops have been determined not to come under USDA’s plant-pest regulations (USDA, 2020) and have been cleared for planting in agroecosystems. Some of these have been gene-edited. For example, seven lines of gene-edited pennycress were reviewed by USDA from 2018–2020 under the old “Am I Regulated”? (AIR) process (before the May 2020 USDA SECURE rule was passed). They were determined to fall outside of USDA’s plant-pest regulations, and although USDA noted some concerns about pennycress being an agricultural weed (USDA, 2018), they were cleared for planting. The USDA’s new SECURE rule grants automatic approval to gene-edited and other GM crops that have already been approved through the AIR process, and many newer gene-edited crops will also be exempt from regulation by USDA under SECURE (USDA, 2020). SECURE will trigger regulatory-review only if gene-edited developers introduce sequences that are not found in that species’ gene pool. Exemptions may also be extended to genes coming from a sexually-compatible species.

Under SECURE, GM and gene-edited crops that are not automatically exempt will enter a screening stage called the Regulatory Status Review (RSR). USDA estimates 99% of GM crops will stop being reviewed by USDA after the RSR (Stokstad, 2020), and these crops would not require a publicly disclosed risk assessment, field trial, or permit (Kuzma and Grieger, 2020; USDA, 2020). Only the estimated 1% that pose a potential plant-pest risk would require a full risk assessment, permit for field trial, or any geographic restrictions. In summary, US regulation may not be the biggest barrier to gene-edited cover-crop development, given the SECURE exemptions and screening process for the RSR. One SMES noted that the GE “*regulatory process [is] much shorter*” in comparison to past GM crop regulation.

However, for some smaller companies and academic producers, the exemptions under SECURE and complex review pathways under SECURE may be difficult to navigate initially (Kuzma and Grieger, 2020). In the interviews, some SMESs expressed concern about regulation as a barrier to gene-edited cover-crop development. One noted “*the regulatory burden of GMOs was so high so that only seed companies with lots of capital can do transgenic events*.” This perception may be due to the high cost estimates (circa $6 to $15M) for regulation of 1st generation GM crops (Kalaitzandonakes et al., 2007) done before the advent of the AIR process and SECURE rule. However, even these cost estimates often included molecular and agronomic characterization and other categories not directly related to safety assessments of GM crops (Kalaitzandonakes et al., 2007; Phillips, 2014). Furthermore, other estimates of regulatory costs for GM crops from public breeders and academics have been significantly less (Schiek et al., 2016). Even though regulatory costs may not be as high as gene-edited cover crop developers anticipate (Lassuoed et al., 2019), small regulatory costs may nonetheless be prohibitive with limited investment in cover crop GE.

Other regulatory-related barriers to gene edited cover crops may be more important than the costs of going through the formal US regulatory system. First, the National Organic Standards Board has decided to exclude gene-edited crops from being certified as organic. This could create issues with the coexistence of organic versus non-organic farmers (such as those planting GE CLCs), cross-contamination through inadvertent comingling or gene flow leading to potential loss of markets for organic farmers, and segregation for different markets. A second related concern is that the EU and other countries have decided to regulate gene-edited crops more stringently and require labeling of gene-edited agricultural food products. With no formal regulation for most gene edited crops in the US and no labeling required for the vast majority of gene-edited foods under the new National Bioengineered Food Disclosure Standards (Jaffe and Kuzma, 2021), it will be nearly impossible to track gene-edited crops through the US food or feed supply (Kuzma and Grieger, 2020). The lack of traceability could create barriers to trade for farmers choosing to grow gene-edited cover crops and thus pose a financial risk from lost markets over concerns about cross contamination or comingled product streams. These concerns on domestic and global market may also make investors view investments in gene-edited crops, including cover crops, as more risky than conventional crops.

#### Political Economy

Developers of gene-edited crops for CLC agriculture require financial capital. However, many factors may limit availability of such capital, creating additional barriers for exploration of these crops. Concern about limited investment in fallow-season cover crops and the political economy of these crops was raised multiple times by the SMESs interviewed. The 1st generation of GM crops was dominated by large commodity crops like corn, soybeans, and cotton that now permeate US agricultural systems at over 93% total acreage (ISAAA, 2018). It was also dominated by large companies selling a high volume of GM crop seeds. In contrast, although cover crops grew in acreage by 50% from 2012–2017, they are incorporated on only 1.7% of US farmland (Runck et al., 2020; Rundquist and Cox, 2021). Cover crops are also usually planted to restore soil health, for weed control, or for other sustainability purposes. Worries about commercial investment in gene-edited cover crops thus seem warranted, given the history of 1st generation GM crops marked by large companies and seed sales’ volume. SMESs whom we interviewed acknowledged the challenges with gene-edited cover crops in that it “*must be financially viable to grow the crop*” and “*cover crops are usually low value and low cost seed, [So] who will make the investment to improve a cover crop [with GE] that will continue to compete with low cost versions of the same crop?*”

Runck et al. (2020) discuss the need for a robust cover-crop seed industry that can provide affordable seed for producers, and they estimate that widespread US cover-crop adoption would require growing cover-crop seed on several million acres of cropland. Thus, cover-crop seed production would necessarily displace a proportion of the production of traditional cash crops. As such, economic incentives for cover crops would be needed, and if the cover crops were gene-edited, these incentives may become even more important to recoup the investment in laboratory R&D to produce them. Cover crops may decrease soil and chemical inputs needed for cash-crop production in alternate seasons and ultimately provide a net economic benefit to farmers, but whether this is enough of a financial benefit is unclear and will be context dependent.

Given these challenges to fallow-season cover cropping, CLC crops that combine sustainability benefits with production of valuable agricultural commodities may be necessary to motivate investment in seed cost for farmers and R&D for seed producers. For example, SMESs mentioned how GE could be used for a low lignin trait in alfalfa for “*happier cows producing more milk while eating less*” and improved “*camelina oil yield or quality … to get farmers a decent economic value proposition.*” These uses would have sustainability benefits to soil as cover crops as well as financial benefits to farmers.

Another economic issue for gene-edited cover crops is centered around ownership and intellectual property (IP). A SMES interviewee noted that “*with regard to I.P. for small companies this [technology] is very expensive. CRISPR patent is held by two groups and that is very expensive each time [for] a license fee*.” Montenegro deWit (2020) also found in her analysis that “despite the opening up of CRISPR IP for non-commercial research, CRISPR’s commercial development remains tightly bound up in patents and licensing agreements.” Another study noted with regard to gene-edited crops that “larger industry players…already appear to be more in control of the technology’s agricultural and food applications” (Egelie et al., 2016). For example, DuPont Pioneer’s gene-edited waxy corn is expected to be released into US markets under standard utility patent restrictions for one-time use (Montenegro de Wit, 2020). Licensing fees to develop gene-edited cover crops for commercial use may be prohibitive for smaller companies or public developers. Patented seeds for gene-edited cover crops could be prohibitively expensive if farmers are not able to commercialize or utilize products from them, in addition to reaping the sustainability benefits.

SMES interviewees summarized political economy concerns as “*producing a line and then introducing the plant in the field needs investment, and for cover crops, if companies do not have much interest they will not work on it, not invest in it.*” Even if companies are interested in investing in GE cover crops “*ownership of these technologies is an issue. It’s dependent on profits, answerable to the shareholders. [So] how to build these technologies for the common good*” *-*- remains an outstanding question.

#### Public Acceptance (“Social License”)

A majority of SMESs (plant breeders and geneticists) interviewed (13 of 17) expressed concern about public perception of gene-edited crops, including cover crops. Several of their comments fall into the “deficit model” thinking of public acceptance and communication. The “deficit model” assumes that a lack of public understanding or knowledge of science has led to the present skepticism toward science—that is, the public is assumed to be “deficient” (of knowledge) while the scientific establishment is “sufficient” (in deserving a lack of skepticism, or in being trusted) (Sturgis and Allum, 2004). One SMES stated*:* “there will always be people who are scared by the technology—so education is as important as the technology”, implying that if the public were educated, they would be less scared and more supportive of gene-edited crops. Other SMESs echoed this view with comments noting the *“really lousy job in introducing and educating public with GMOs”; that “a lot of social license restrictions [are] due to people not understand[ing] how the GM science works”; or that the real problem with the public was “their lack of knowledge*”. Some dismissed public concerns about gene-edited crop risks as *“conspiracy theory is everywhere and people can create a climate of fear”.*

However, social science studies of public attitudes towards GM crops have shown that knowledge has modest and variable effects on public acceptance, with both positive and negative effects observed across multiple studies (Rose et al., 2019). In fact, researchers found those with higher levels of perceived familiarity are more concerned with GM foods, contradicting a main premise of the “deficit model” (Rose et al., 2019). In addition, other factors seem to be more important for public perception of GM crops such as trust in scientists and governments to manage risk, legitimacy of decision processes, respect for diverse cultural values and world views, and the public’s ability to control their own exposure to risk or make their own choices about technological products (e.g. Siegrist et al., 2012; Yue et al., 2015a; also reviewed in; Kuzma, 2017).

In a recent public perception study comparing gene-edited to GM and conventional foods, researchers found that respondents viewed CRISPR and GM food similarly and substantially less positively than conventional food (Shew et al., 2018). The authors state that their study does not bode well for consumer acceptance of gene-edited foods. It is possible however that certain benefits can outweigh negative perceptions of GM and potentially gene-edited foods among some consumers, and there is support in the literature for the positive impact of specific benefits such as health, safety, and nutrition (Siegrist, 2008; Yue et al., 2015b). Several interviewees did mention that “convincing the public that these crops are beneficial” may help with public acceptance, but caution should be warranted with the attitude that “convincing” the public is the right approach. In light of other technological risk perception factors, engaging consumers and equipping them with information and choice seem better approaches to engendering trust and reducing skepticism towards gene-edited crops and foods.

Importantly, the possibility of public rejection of gene-edited crops creates major risks for all parties with a direct economic stake in their development. Crop developers and investors risk loss of financial capital and opportunity costs associated with development of gene-edited crops; advocacy organizations might lose reputational or political capital by endorsing GE crops that are subsequently rejected by the public. This complex of risk complicates the political economy of these crops.

The concept of “social license”—a notion borrowed from the mining industry—was mentioned by several interviewees and viewed to be important for the success of gene-edited cover crops. However, the utility of social license in biotechnology policy has been criticized, because the concept entails a limited scope of public engagement (Delborne et al., 2020). Social license implies that scientists and decision makers need only to ask for public permission once (a license) after technologies have already been defined and assessed by expert communities (Delborne et al., 2020). In contrast, meaningful public engagement would include stakeholders and publics in the formulation of problems to address with GE, in defining endpoints for risk assessment, and in continual monitoring and re-evaluation of gene-edited products in the face of uncertainties and complexities of releasing them into agroecosystems. One SMES suggested a solution closer to meaningful public engagement by stating that *“a collective/broad group could develop a scale on which individual [GE] technologies could be weighed to see how they effect a community”.* Calls for community engagement in GE and decision making have been made by several researchers and scholars (e.g., Jordan et al., 2017; Kofler et al., 2018; Kuzma and Grieger, 2020).

### Current Approaches to Governance

Unfortunately, gene-edited crop developers are repeating mistakes in governance that occurred with the 1st generation of GM crops and foods, which may increase risk and costs associated with the use of GE to advance crops for CLC agriculture.

First, developers continue to take somewhat contradictory stances and make unsubstantiated claims about the technology and regulation (Kuzma et al., 2016; Kuzma, 2018; Bain et al., 2020) that public critics have recognized and critiqued in the past. Developers tend to communicate that although GE is a phenomenal technological leap that shows great promise, it is nothing new in comparison to conventional breeding and should therefore not undergo regulation (Kuzma et al., 2016; Kuzma, 2018; Bain et al., 2020; Qaim, 2020). This hypocrisy has been detected and noted by various publics in past controversies. Also, overpromising that GE is necessary for a second green revolution (Bain et al., 2020) may engender public mistrust, as 1st generation GM crops did not appreciably increase yields on average (Gould et al., 2017).

Second, consumers generally want to know that technological products are being regulated and the scope of governance includes potential health and environmental risks. Yet, many gene-edited crop developers have taken the stance that these crops should not be regulated. The lack of oversight, or a failure to minimize harm (e.g., USDA will not screen for off-target edits or regulate based on weediness risks Kuzma and Grieger, 2020), may jeopardize public confidence.

Third, as regulatory processes are developing in the US, it appears that there will be a lack of transparency about what gene-edited crops are being reviewed and how they are regulated (Kuzma, 2018; Kuzma and Grieger, 2020). In addition, most gene-edited foods will not be labeled (Kuzma, 2018). Without labeling, consumers do not have access to information to make their own informed decisions, which takes control away from them in determining their own exposure to risks, however small they may be. Ample risk perception studies indicate that people view risks they cannot control as higher than those they can (e.g., Slovic, 1987). There are also efforts to obfuscate terminology—new USDA labeling standards do not use the term GM (instead use bioengineered) and gene-edited crop developers use terms like “new plant breeding technology” (NPBT). With the 1st generation of GM foods, consumers were largely unaware that they were eating them for years, and now there is a concerted backlash against them in the marketplace as organic and non-GM markets grow. Consumers may feel tricked by differential terminology and the lack of transparency, should they be able to find out that GE is a derivative of modern biotechnology, and trust in GE industries would be difficult to restore (Kuzma, 2018).

Finally, our regulatory system for gene edited crops is based on a narrow set of direct health and environmental risks. Yet, consumers care also about indirect ecosystem risks and benefits (e.g., climate change or resource use); health risks such as food allergenicity or sensitivity from low level consumption over a lifetime; social, economic, and cultural impacts; procedural justice and social equity; respect for nature; and ethical dimensions of rights to know and choose. For decades with GM crops, these broader societal aspects were marginalized and there is no space for legitimate discussion of them as scientists were adamant about “science-based regulation” (which almost exclusively addressed direct health or environmental risks that could be shown in laboratory-based toxicity studies). There has been a lack of respect for concerns voiced that are outside of the narrow purviews of the regulatory agencies (Thompson et al., 2007; Kuzma, 2018). Power and voice are given to a narrow set of technical experts, largely those of the product developer and regulatory staff (as public federal advisory committee processes have been lacking recently for gene edited products) (Kuzma, 2018). Yet, procedural fairness is an important factor for public acceptance of GM crops (Siegrist et al., 2012).

In summary, systemic barriers to exploration of gene-edited crops for CLC agriculture are being created by the regulatory landscape, political economy, public acceptance, and current governance approaches to gene-edited crops. If these barriers are not proactively addressed, we suspect these barriers will greatly slow exploration of these crops. Below we discuss responsible innovation and governance models that may enhance public trust, procedural legitimacy, and public confidence in gene-edited cover crops, and which may therefore be necessary adjuncts to the GE technologies themselves.

### Shared Governance and Robust Risk Management: Key Support Pillars for Development of New Crops for CLC Agriculture?

In this section, we draw on insights from the CG pilot project and literature, but also our experience with GM crop governance over the past 30 years, observations of the field, and normative conclusions from these observations and experiences. We propose that societal adoption and acceptance of gene-edited crops for CLC agriculture—if these are to occur—may require replacing outdated notions of “deficit model thinking” and “social license” with more collaborative and publicly-robust governance processes.

Towards this end, a range of models for governance of gene-edited crops have been proposed. One example, as noted previously, is the Jordan et al. (2017) Cooperative Governance model for gene-edited cover crops. This model, piloted in the current project, engages a network of multiple subject matter experts, stakeholders, and investors in decision making about whether to move forward with a gene-edited cover crop. The multi-stakeholder cooperative governance group would conduct a comprehensive, multi-criteria assessment of the relative risks and benefits of a gene-edited crop designed for a specific purpose or environment. The group would also consider societal, economic, and cultural aspects before deciding to move forward with the gene-edited cover crop. Investors would mitigate risk by investing in crops that underwent such a rigorous evaluation by the diverse group.

Kuzma and Grieger (2020) recently proposed a “community-led and responsible governance” (CLEAR-GOV) model for gene-edited crops that would center on a repository of information about what gene-edited crops enter agroecosystems and food markets and a certification process to incentivize the sharing of such information. They note the lack of public information for many exempt crops under the new SECURE rules and the lack of labeling and traceability in discussing the need for such a repository. A multi-stakeholder advisory group, in concert with a public advisory group and crop-developer input, would guide the information required to be certified, the structure of the repository and the balancing of public information with IP protection and privacy. At a minimum the host plant, growth environment, purpose of the trait, and potential uses should be deposited. They argue that such transparency is more likely to engender public and consumer trust.

Some developers of gene–edited crops are working with the non-profit Center for Food Integrity (CFI) and an associated multi-stakeholder coalition to draft guidelines for responsible use of GE (The Centre Food Integrity, 2020). This model entails voluntary stewardship certification, intiated by GE developers self-assessing themselves against a checklist of best practices; a verification group then reviews self-assessments. Developers would have discretion to conceal edited plant varieties and traits as confidential business information, and no central repository of gene-edited crops and traits would be maintained.

Finally, Kuzma (2019) described a more open and “procedurally robust” risk assessment framework for transgenic organisms. This framework highlights that risk analysis is laden with assumptions and interpretations based on values. For example, the endpoints chosen in a risk assessment are based on what involved stakeholders care about (e.g., certain species, certain products of agriculture, or certain natural resources, etc.). Science gives us a guide, but what risks are acceptable are based on values, taking into consideration particular experiences, culture, perceptions of benefits, control over the situation, and trust in those managing risks (Kuzma, 2017). When new biotechnology products are initially released into ecosystems, evaluating the “substantive validity” of risk assessments –where outcomes of the risk assessment are compared to what happens in reality—is not generally feasible, especially prior to any environmental release. Therefore, “procedural validity” of the risk assessment—i.e., how the risk assessment is conducted—becomes even more important than attempting to ascertain the substantive validity of particular risk evaluations prior to initial release and monitoring.

Following this reasoning, Kuzma (2019) outlined a framework for conducting robust risk analysis in support of formal regulatory decision making: the “Procedurally Robust Risk Analysis Framework” (PPRAF). The framework draws upon “responsible innovation” principles of humility, procedural validity, inclusion, anticipation, and reflexivity. PPRAF call on risk analysis to acknowledge uncertainty, engage multiple interested and affected parties in a holistic discussion of ends and means of innovation and associated risks; anticipate future conditions and contigencies; and promote mutual learning and reflection on the transparency, openness, and procedural validity of the risk analysis, and of uncertainty associated with conclusion.

The above governance and risk assessment models cannot guarantee public acceptance, but they are more likely to engender legitimacy and trust. Trust in government or experts to manage technologies has been a factor identified as a key factor for public acceptance of technologies (e.g., Siegrist et al., 2012; Yue et al., 2015a; Yue et al., 2015b).

### Prospects for Implementation of Robust and Responsible Governance and Risk Assessment Models

Our deliberative workshops identified a moderately broad shared interest, among a multi-sector group, in several potential models for robust and responsible governance and risk assessment. Two of these models can be outlined as follows.

Stakeholder Governance: Deliberative foresight assessment by a broad and diverse range of stakeholders to evaluate social, scientific, economic, and cultural impacts, both positive and negative, of gene-edited crops. Crop developer decides on crop release. The crop would be certified for “inclusive stewardship.”

Community Governance: Deliberative foresight assessment to evaluate crops by a broad and diverse range of interested and affected people, e.g., community groups including marginalized and indigenous communities and organic farmers. Consensus or majority decision regarding crop release. The crop would be certified for “responsible development and community approval.”

However, there was a considerable range of opinion about the merits of these models, and some robust disagreement during the deliberative process. We also note that biotechnology industry innovators (Roberts et al., 2020) were found to be skeptical of the practicality of such responsible innovation and governance methods, particularly on the basis of perceived time demands and concern that such methods will make the innovation process too slow. Consequently, we recommend an exploratory application of these method in a particular pilot situation, and are pursuing that in current stages of our pilot governance project.

## Conclusion

We found that a group of plant breeders developing cover crops and other crops for enhancing productive living cover in agriculture are very eager to use GE as a tool for developing CLC crops. A group of agroecologists working on development of diversified agroecosystems are strongly committed to enhancing CLC agriculture, and see merit in applying GE as a tool for developing relevant crops. However, the agroecologists also have a number of concerns about potential environmental consequences of applications of GE. Generally, these consequences are typical of agronomic and agroecological effects that can accompany any diversification of an agroecosystem, e.g., new pest problems, potential escape of invasive feral populations of a new crop, or changes in nutrient cycling.

Like plant breeders and agroecologists, other participants in the cooperative governance pilot projects also expressed generally positive views of GE as a means of developing new crops for diversification of agriculture. However, project participants from many sectors have concerns about societal impacts of applications of GE. These concerns center on procedural and distributional justice issues—who will govern the applications of GE, and how will benefits resulting from successful development and scaling of these crops be distributed? Likewise, how will costs and risks associated with the scaling of these crops be distributed. How will these applications of GE be governed, and what groups or parties will have power and influence in governance? More broadly, what kind of agriculture will result from applications of GE?

Broadly, it appears that responsible innovation and scaling practices and approaches will be necessary to address these concerns. In essence, the current situation features many social factors that pose challenges to applications of GE to advance CLC crops. First, the regulatory landscape is complex, varying markedly across global regions, and creating dilemmas and moral hazards for crop developers that may strongly limit the development, adoption, and use of GE CLC crops. Secondly, there are significant political-economic barriers to development of CLC crops, which will take concerted cross-sector action to surmount. Thirdly, current governance and risk-management approaches risk triggering strong opposition by civil-society groups. Sustained use of responsible innovation and scaling practices and approaches may surmount these barriers. At present, the willingness of a broad range of societal actors to participate in sustained responsible innovation and scaling processes is very unclear. Relevant actors have little experience with responsible innovation and scaling approaches, and therefore additional pilot projects are urgently needed.

The need for additional piloting of responsible innovation and scaling is particularly urgent because the current status quo may drive a dynamic of increasing uncertainty and opposition to use of GE, amplified by the stances of powerful food system actors such as CPG firms, which currently appear largely unwilling to publicly discuss potential applications of GE. If continued, we expect that this dynamic will greatly inhibit investment and exploration of GE for development of CLC agriculture or other forms of diversified agriculture. Specifically, to advance and scale, crops for CLC agriculture must attract the “innovation accelerators” highlighted by Herrero et al. (2020), including finance, supportive policy, markets, ongoing R&D, and concerted cross-sector collective action, to both advance and scale these crops and to govern their development. If the status quo continues, emerging CLC crops appear unlikely to attract the requisite innovation accelerators or pillars of support needed have impact at scale and to reward public and private investment.

In closing, we suggest that is it is now essential to approach GE not as a standalone innovation, but rather as an element of “socio-technological innovation bundles” (Barrett et al., 2020). Such “bundles” are systems that include GE coupled variously with other relevant innovations that are broadly social in nature, e.g., in responsible innovation and scaling, and perhaps in other aspects, such as novel cooperative business and crop stewardship structures (Gary, 2019) and finance innovations such as Environmental, Social, and Governance Investing (ESG, Tucker and Jones, 2020). In our view, GE coupled to responsible innovation and scaling and other innovations appears to have high potential to attract broad societal support and could be applied widely in development of new crops to address agricultural diversification and related grand challenges. In the absence of such coupling, such use of GE may entail larger risks for crop developers and encounter strong societal opposition.

## Data Availability

The raw data supporting the conclusion of this article will be made available by the authors upon request, without undue reservation.
